# Antiviral Activity of Fermented Ginseng Extracts against a Broad Range of Influenza Viruses

**DOI:** 10.3390/v10090471

**Published:** 2018-09-01

**Authors:** Ye Wang, Yu-Jin Jung, Ki-Hye Kim, Youngman Kwon, Yu-Jin Kim, Zhan Zhang, Heun-Soo Kang, Bao-Zhong Wang, Fu-Shi Quan, Sang-Moo Kang

**Affiliations:** 1 Center for Inflammation, Immunity and Infection, Institute for Biomedical Sciences Georgia State University, Atlanta, GA 30303, USA; ywang91@student.gsu.edu (Y.W.); yjung8@student.gsu.edu (Y.J.J.); kihyekim4282@gmail.com (K.H.K.); ymankwon@gmail.com (Y.K.); yujinsm@gmail.com (Y.-J.K.); zzhang12@student.gsu.edu (Z.Z.); bwang23@gsu.edu (B.-Z.W.); fquan01@gmail.com (F.-S.Q.); 2 Department of Biology, Georgia State University, Atlanta, GA 30303, USA; 3 Metabolab, Cancer Research Institute, Seoul National University College of Medicine, Seoul 110-799, Korea; metabolab@gmail.com; 4 Department of Medical Zoology, Kyung Hee University School of Medicine, Seoul 130-705, Korea

**Keywords:** fermented ginseng, influenza virus, antiviral activity, hemagglutinin, neuraminidase

## Abstract

Ginseng products used as herb nutritional supplements are orally consumed and fermented to ginsenoside compounds by the intestinal microbes. In this study, we investigated antiviral protective effects of fermented ginseng extracts against different strains of influenza viruses in genetically diverse mouse models. Intranasal coinoculation of mice with fermented ginseng extract and influenza virus improved survival rates and conferred protection against H1N1, H3N2, H5N1, and H7N9 strains, with the efficacy dependent on the dose of ginseng samples. Antiviral protection by fermented ginseng extract was observed in different genetic backgrounds of mice and in the deficient conditions of key adaptive immune components (CD4, CD8, B cell, MHCII). The mice that survived primary virus inoculation with fermented ginseng extract developed immunity against the secondary infection with homologous and heterosubtypic viruses. In vitro cell culture experiments showed moderate virus neutralizing activity by fermented ginseng extract, probably by inhibiting hemagglutination and neuraminidase activity. This study suggests that fermented ginseng extracts might provide a means to treat influenza disease regardless of virus strains.

## 1. Introduction

Influenza virus is a contagious pathogen that has been causing massive mortality for decades. Among the three major types (type A, B, and C) of influenza virus, type A is more prevalent due to diverse hosts. The Center for Disease Control and Prevention (CDC) estimates that influenza has resulted in between 12,000 and 56,000 deaths annually in the United States since 2010 [1,2]. Two important glycoproteins—hemagglutinin (HA) and neuraminidase (NA)—determine a specific subtype and mediate infection. With a combination of HA (subtype 1 to 18) and NA (subtype 1 to 11), various subtypes of influenza virus can infect different species, including humans, mammals, and birds [3,4,5]. Among these subtypes of influenza virus, H1N1 and H3N2 are the main strains circulating in humans every season, whereas sporadic transmission and infection with novel H5N1, H7N9, and H5N6 subtypes in humans have been reported recently [6,7,8]. Vaccination is the main measure used to prevent large-scale infection. However, influenza viruses continue to mutate their genomes, resulting in frequent and high resistance to drugs and vaccines [9,10].

Panax ginseng has long been reported as one of the most common herbal medicines used in humans. Treatment of mice with ginseng extracts has been reported to reduce the production of inflammatory IL-6 and IL-8 cytokines and to increase antiviral cytokine interferon (IFN)-γ upon influenza virus infection [11,12]. Ginseng products are mostly consumed by oral intake. During the digestion process, ginseng ginsenoside compounds (Rg1, Rb1, Rb2, Rg3) are converted into pharmaceutically active components (PPD, Rh2, Compound K, PPT) by bacterial microbes present in human intestines [13]. Fermented ginseng products have been shown to exhibit various biological activities, including antioxidative and antibacterial activities [14], alleviation of the severity of dextran sulfate sodium-induced colitis [15], and antidiabetic effects [16]. However, it has not been well known whether fermented ginseng extracts have antiviral activity, conferring in vivo protection against influenza virus.

In this study, we investigated antiviral effects of fermented ginseng extract samples on influenza viruses. Results in this study demonstrated that fermented red ginseng extracts had an antiviral protective activity against influenza viruses from different subtypes (H1N1, H3N2, H5N1, H7N9) in in vivo mouse models. In vitro cell culture studies suggested mechanistic insights into the antiviral activity of the fermented ginseng extracts. Fermented ginseng products showed higher in vivo antiviral effects against influenza viruses compared to nonfermented ginseng samples.

## 2. Materials and Methods 

### 2.1. Cells, Viruses, and Ginseng Samples

Madin–Darby canine kidney (MDCK) cells purchased from ATCC (Manassas, VA, USA) were cultured in Eagle’s Minimum Essential Medium (EMEM) and used for microneutralization tests and plaque assays. Subtypes A/Philippines/82 (H3N2), A/California/04/2009 (H1N1pdm), and A/WSN/1933 (H1N1) viruses were kindly provided by Dr. Huan Nguyen, Dr. Richard Webby, and Dr. Yumiko Matsuoka, respectively. The reassortant A/Vietnam/1203/2004 virus (A/VN1203), reverse genetic recombinant A/VN1203, rgH5N1 containing HA with polybasic residues removed and NA from A/VN1203, and six internal genes from A/PR/8/1934 (H1N1) was described in our previous study [17]. The reassortant rgH7N9 virus was generated to contain H7 HA and N9 NA genes from A/Shanghai/2013 and six internal genes from A/PR/8/1934 backbone virus using reverse genetics. The H7 HA and N9 NA genes were kindly provided by Dr. García-Sastre. These influenza viruses were propagated in 10-day-old embryonated eggs [18]. The egg allantoic fluids were harvested and stored at −80 °C until use. The fermented ginseng extract samples used in this study were obtained from Metabolab Co., Ltd. (Seoul, South Korea). Briefly, the preparation of the fermented ginseng extract samples was carried out by a complex fermentation procedure combined with patented strains of *Lactobacillus alimentarius* M-2 (KCTC 11054 BP), *Leuconostoc mesenteroides* M-3 (KCTC 11055 BP), and pectolytic enzymes (Novozymes, Kobenhavn, Denmark) for 7 days (Korean Patent No. KR-1008774890000). The fermentation for preparing fermented ginseng sample A (F.G.A) was processed for the extended time of 7 days for high levels of compound K production, whereas the fermentation time for fermented ginseng sample B (F.G.B) was shortened for 2 days so that the ginsenoside components (Rb1 + Rg1) would be retained above a level of 2.5 mg/g of ginseng sample. The condition of strains and enzymes was identical for preparation of F.G.A and F.G.B. The components of fermented ginseng extract samples A and B are presented in Table 1. The commercial nonfermented ginseng sample was kindly provided by Korea Ginseng Corporation (Daejeon, Korea). Briefly, fresh roots of the *Panax ginseng* were washed, steamed at 100 °C for 2–3 h and dried. The dried red ginseng roots were boiled in 4–5 volumes of water for 3 h, and the supernatant ginseng extracts were concentrated and manufactured for nonfermented ginseng extract products.

### 2.2. Mice and Treatment

Wild-type mice (BALB/c and C57BL6) and mutant mice (CD4 T cell-deficient B6.129S6-Cd4^tm1Knw^/J, CD8 T cell-deficient B6.129S2-*Cd8atm1Mak*/J, B cell-deficient µMT B10.129S2(B6)-*Ighmtm1Cgn*/J, MHCII deficient (B6.129S2-*H2dlAb1-Ea*/J)) were purchased from the Jackson Laboratory (Bar Harbor, ME, USA). To determine in vivo antiviral effects, 25 µL of different strains of influenza viruses were mixed with 25 µL of fermented ginseng extract samples A or B and then incubated for 30 min at 37 °C. The amounts of ginseng samples in the mixtures were in the range of 50–1000 µg doses per mouse. Then, 50 µL of virus–ginseng mixtures were intranasally inoculated into naive mice [19,20]. For the experiment involving treatment with ginseng after infection, wild-type BALB/c mice were infected with A/WSN (H1N1) influenza virus (1 × LD_50_) first. After 1 h postinfection, 1 mg dose of fermented ginseng sample A was intranasally inoculated into the infected mice every hour for five times. 

Body weight changes and survival rates of mice were monitored daily after infection. All animal experiments were followed by the instruction of the Institutional Animal Care & Use Committee (IACUC) of Georgia State University (approved protocol IACUC protocols A14025 on 20 October 2014 and A18001 on 18 September 2017).

### 2.3. Lung Viral Titers, Histology and Cytokine ELISA

Mice were sacrificed at day 6 postinfection with A/Vietnam/1203/2004 (rgH5N1) and bronchoalveolar lavage fluids (BALF), and lung samples were collected from the individual mouse as previously described [21]. Part of the lung samples was fixed with 10% formalin for 24 h, transferred to the serial of 70–100% ethanol, and embedded in paraffin. The sections of lung samples were stained with hematoxylin and eosin, and pictures were taken using a microscope (PLYMPUS VS-120 with VISIOMORPH and TISSUNORPH software, Pittsburgh, PA, USA). Dilutions of lung extracts were inoculated in the 11-day-old embryonated chicken eggs. Lung viral titers were determined by measuring hemagglutination activity and calculated using the Reed–Muenchen method. After 2 days of incubation, allantoic fluids (50 µL) were collected from the eggs infected with each dilution and mixed with 50 µL of chicken red blood cells. The endpoint dilutions were determined after observation of forming the agglutination on the bottom of the V-shape 96-well plates. Levels of cytokines, such as IL-6 and TNF-α in BALF and lung extracts, were determined by ELISA as described [11]. To determine the effect of F.G.A on interaction between antibodies and virus, the 96-well plates were coated with inactivated A/2009/California (H1N1) or A/2004/Vietnam (H5N1) influenza virus overnight, then incubated with 10 mg of F.G.A or nonfermented ginseng for 1h at 37 °C. Anti-H1 HA (NR-19866 BEI resource) and Anti-H5 HA (NR-13449 BEI resource) were used as primary antibodies, and the secondary goat anti-mouse IgG (SouthernBiotech CAT NO 1033-05, Birmingham, AL, USA) was used for the ELISA test.

### 2.4. Virus Microneutralization and Plaque Assays and Cell Viability Tests in MDCK Cells

Virus microneutralization assay using MDCK cells was performed as previously described [22]. In brief, influenza virus was mixed with ginseng extract samples, and the mixture was incubated for 1 h at 37 °C. Then, the virus–ginseng mixture was added to (1 × 10^5^) MDCK cells and cultivated for 18–22 h at 37 °C with 5% CO_2_. After discarding the supernatants, the mixtures with MDCK cells were fixed to the 96-well plate with 80% acetone for 10 min. For the ELISA OD value determination, the primary antibody (anti-NP mouse monoclonal, Chemicon #MAB8257B, Fisher Scientific, Hampton, NH, USA) and the secondary antibody (goat anti-mouse IgG HRP-conjugated, Southern Biotech #7100-05) were used. Virus control wells (VC: virus only) and cell control wells (CC: cells only) were set for OD value calculation: “X” = ((Average of VC wells) − (Average of CC wells))/2 + (Average of CC wells). All OD values below “X” were considered as a positive (virus negative) for the neutralizing activity. TCID_50_ (50% tissue culture infective dose) was used to measure the cell infection rate.

For the plaque forming assays, A/California/2009 (H1N1) and A/Vietnam/1203/2004 (rgH5N1) were mixed with different ginseng samples at a final concentration of 10 mg/mL for 30 min at 37 °C. Then, the mixture was incubated with MDCK monolayer cells for 1 h at 37 °C. After the incubation for 1 h, the virus–ginseng mixture was discarded and the MDCK monolayer was covered with 1.5% agarose with 2× DMEM and trypsin. The whole MDCK monolayer with agarose was cultivated for 3–5 days at 37 °C with 5% CO_2_ and fixed with 4% formalin for 1 h after removing the agarose layer [19]. Crystal violet staining solution is often used in such experiments, but it was not required to observe plaque formation. After drying the 6-well plates, plaques were visible and counted.

For cell viability test, 2 mL of EMEM medium was mixed with different concentrations of F.G.A to make final concentrations of F.G.A to 5 mg and 10 mg/mL. The mixtures were incubated with MDCK monolayer for 6 h. The medium–ginseng mixture was then removed, and cells were washed out by 0.25% (*w*/*v*) trypsin/0.53 mM EDTA. Viable cells were counted after staining with 0.4% trypan blue solution.

### 2.5. Hemagglutinin Assay

Hemagglutination titrations of A/California/2009 (H1N1) and A/Vietnam/1203/2004 (H5N1) strains were determined using a standard method [23]. Fermented ginseng extract samples (25 µL) were mixed with 25 µL of 4–8 HA units of virus at 37 °C for 30 min. The virus–ginseng mixture was then mixed with 50 µL 0.5% chicken red blood cell and incubated for 1 h at room temperature. A negative control (virus only) and a positive control (no virus) were set in this assay.

### 2.6. Neuraminidase Assay

NA-specific inhibition activity by enzyme-linked lectin assays using virus substrates has been well reported in previous studies [24,25,26,27]. Fetuin proteins (Sigma, St. Louis, MO, USA, cat# F3385) were coated on the 96-well plate. Before adding viruses to the plate, 2× virus (25 µL) was mixed with a 2× ginseng sample (25 µL) and incubated at 37 °C for 30 min. The virus–ginseng mixture was incubated in the plate for 2 h. After washing the unbound viruses, HRP conjugate of lectin from Arachis hypogaea peanut (Sigma, cat# L6135-1MG) was added and then incubated for 2 h. *O*-phenylenediamine dihydrochloride (OPD) (Sigma, cat# P8287) was used to check the color development, and plates were finally read in 450 nm after stopping the reaction.

### 2.7. Statistical Analysis Methods

A two-tailed student’s *t*-test was used to determine whether the two different groups showed statistical significance. When *p* value was less than 0.05, it was considered as significant.

## 3. Results

### 3.1. Fermented Ginseng Samples Exhibit Higher Antiviral Protection against Influenza Virus than Nonfermented Ginseng

Orally taken ginseng supplements are digested and fermented in the intestines, producing active ingredients such as ginsenosides, including compound K. We tested whether fermented ginseng samples would have antiviral in vivo protection against a lethal dose of influenza virus infection. The red ginseng extracts were in vitro fermented with a mixture of bacteria and enzymes, and the product compounds were characterized as shown in Table 1. In vitro fermentation of ginseng samples generated new ginsenoside saponin compounds (F1, F2, PPT, CK, Rh2, PPD) but resulted in lower levels of total ginsenosides (Table 1). The fermented ginseng sample A (F.G.A) contained higher levels of new ginsenoside compounds (F1, F2, PPT, CK, Rh2, PPD) compared to those in the fermented ginseng sample B (F.G.B) (Table 1).

**Figure 1:**
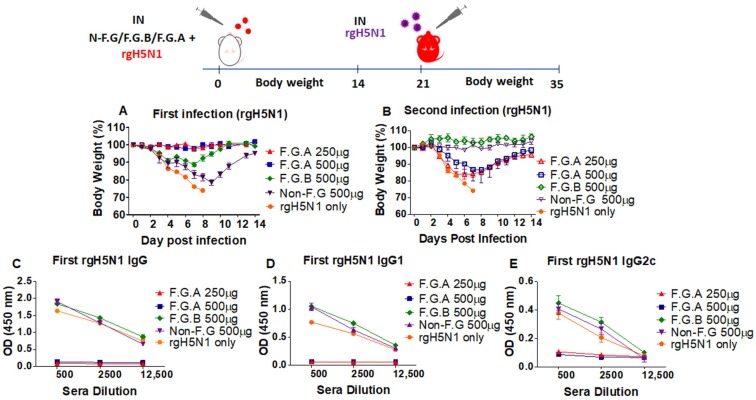
Fermented ginseng sample A showed higher antiviral protective activity against influenza viruses. Groups of mice (*n* = 5, wild-type BALB/c mice) were intranasally (IN) infected with a mixture of 1.5 LD_50_ A/Vietnam/1203/2004 (rgH5N1) and fermented ginseng sample A or B at different doses (250 μg and 500 μg). After 14 days of body weight monitoring, the serum sample was collected to detect the levels of IgG and IgG isotypes using a ELISA method. The mice that survived from the first rgH5N1 infection were challenged again with a lethal dose of rgH5N1 (3 LD_50_). (**A**) Body weight change after the first infection (rgH5N1) and (**B**) body weight change after the second infection (rgH5N1). (**C**) rgH5N1-specific IgG, (**D**) IgG1, and (**E**) IgG2a after the first infection. F.G.A: rgH5N1 virus + fermented ginseng sample A; F.G.B: rgH5N1 virus + fermented ginseng sample B; non-F.G: not fermented ginseng; rgH5N1 only: virus infection without ginseng samples.

To test the antiviral protective effects of the fermented ginseng samples A and B, groups of mice (wild-type BALB/c) were intranasally infected with a lethal dose (1.5 LD_50_) of rgH5N1 virus alone or rgH5N1 virus mixed with the fermented ginseng extracts of A or B (F.G.A or F.G.B) or nonfermented ginseng (non-F.G) extract. High and moderate doses (500, 250 µg) of F.G.A and high dose (500 µg) of F.G.B or non-F.G were first tested (Figure 1). In the group with F.G.A, at doses of either 500 µg or 250 µg, none of the mice showed weight loss after infection with rgH5N1 virus (F.G.A 500, 250 µg, Figure 1A). However, in the group with fermented ginseng B or nonfermented ginseng (500 µg), all mice began to lose weight from day 3 postinfection (F.G.B, Non-F.G, 500 µg, Figure 1A). From day 7 or day 9, all mice from fermented ginseng B or nonfermented ginseng groups began to recover, respectively, while all mice in the rgH5N1 virus-only infection group died by day 8 postinfection (Figure 1A).

Viral replication during the primary infection of mice induces immune responses of antibodies and cross protective T cells. Protection by inhibiting complete viral replication does not induce virus-specific immune responses. Next, we examined whether the infected mice generated rgH5N1-specific antibodies. We collected the immune sera from all mice from the first rgH5N1 virus infection and determined the induction of antibody responses due to the viral replication. The groups of mice with F.G.B, non-F.G (500 µg), and naive mice that survived from a sublethal dose of rgH5N1 virus infection showed high levels of virus-specific antibody responses due to influenza viral replication. By contrast, the fermented ginseng sample A groups (F.G.A 500, 250 µg) showed no significant virus-specific antibodies, suggesting complete protection against viral replication (Figure 1C,D,E).

We then applied the secondary infection with 3 LD_50_ of rgH5N1 to those groups that survived from the first infection (Figure 1B). Due to the complete protection during the primary infection by the antiviral activity of F.G.A, no antibody responses were produced and the mice that survived the virus mixture of F.G.A (500, 250 µg) showed significant body weight loss (15–20%) during the secondary infection; this indicated complete protection only during primary infection. Nonetheless, compared to the naïve infection group (Figure 1B, naïve rgH5N1 only), all the mice from F.G.A (500, 250 µg) survived and showed less body weight loss. Meanwhile, the mouse F.G.B and nonfermented ginseng (500 µg) groups did not display body weight loss. As expected, from the levels of rgH5N1 virus specific antibodies and disease of weight loss during the primary virus infection, these mice (F.G.B 500 µg, non-FG 500 µg) developed strong immunity.

### 3.2. Protected Mice with Fermented Ginseng and rgH5N1 Influenza Virus Inoculation Develops Immunity against Virus Infection in Future

**Figure 2:**
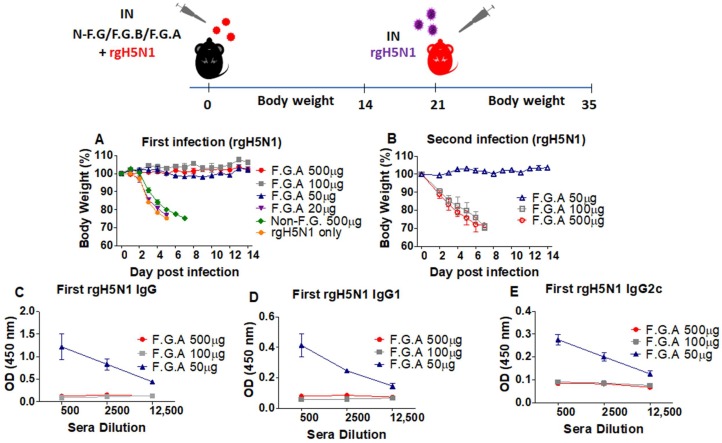
Low dose of fermented ginseng extract sample A provided protection against rgH5N1 influenza virus during primary and secondary infection. Groups of mice (*n* = 5, wild-type C57BL/6) mice were intranasally (IN) infected with a mixture of 10 LD_50_ of A/Vietnam/1203/2004 (rgH5N1) virus and fermented ginseng sample A or B at different doses (20, 50, 100, 500 μg). After 14 days of body weight monitoring, the serum sample was collected to detect the levels of IgG and IgG isotypes using an ELISA method. The mice that survived from the first infection were challenged again with rgH5N1 (10 LD_50_). (**A**) Body weight change after the first infection (10 LD_50_, rgH5N1) and (**B**) body weight change after the second infection (15 LD_50_, rgH5N1). (**C**) rgH5N1-specific IgG, (**D**) IgG1, and (**E**) IgG2c after the first infection. F.B.A: rgH5N1 virus + fermented ginseng sample A; F.G.B: H5N1 virus + fermented ginseng sample B; rgH5N1 only: virus infection without ginseng samples.

Since the fermented ginseng extract sample A showed better antiviral protection activity, we further investigated whether a lower dose of sample A would exhibit such an antiviral effect without weight loss and acquire immunity against secondary infection. In this set of experiments, a different genetic background of C57BL/6 mice was used to test in vivo antiviral activity of ginseng samples. Different doses of F.G.A (100, 50, and 20 µg) with a high dose of rgH5N1 virus were applied to the wild-type C57B/6 mice (*n* = 5). As a positive control, a high dose (500 µg) of F.G.A was also included in this experiment. Moderate to low doses (100 and 50 µg) of F.G.A showed 100% protection without displaying weight loss, as observed in the high dose (500 µg) F.G.A group. However, the lowest dose (20 µg) of F.G.A did not show any antiviral protective effect against the high dose (10 LD_50_) of rgH5N1 virus (Figure 2A, F.G.A 20 µg). As a negative control, mice from the group of nonfermented ginseng also died at day 7 postinfection with rgH5N1 virus (Figure 2A, non-F.G 500 µg).

As in the 500 µg F.G.A group, the treatment with mixture of 100 µg F.G.A and rgH5N1 virus (10 LD_50_) did not induce virus-specific antibodies (Figure 2C,D,E) during infection, suggesting complete inhibition of viral replication and protection. As expected, the mice from these 100 µg and 500 µg F.G.A groups that survived during primary infection were not protected during the secondary infection (Figure 2B). However, the dose of 50 µg F.G.A-treated mouse group showed protection without body weight loss (Figure 2A), induced rgH5N1 virus-specific antibodies during primary infection (Figure 2C,D,E), and was protected without weight loss during the secondary infection (15 LD_50_ H5N1, Figure 2B). These results suggest that the F.G.A protected the mice against influenza virus in a dose-dependent manner and that treatment with an optimal dose of fermented ginseng and virus provides protection against primary infection and induces immunity against secondary infection.

### 3.3. Fermented Ginseng Sample Has Antiviral Protective Effects against H3N2 Influenza Virus in CD8-Deficient Mice

**Figure 3:**
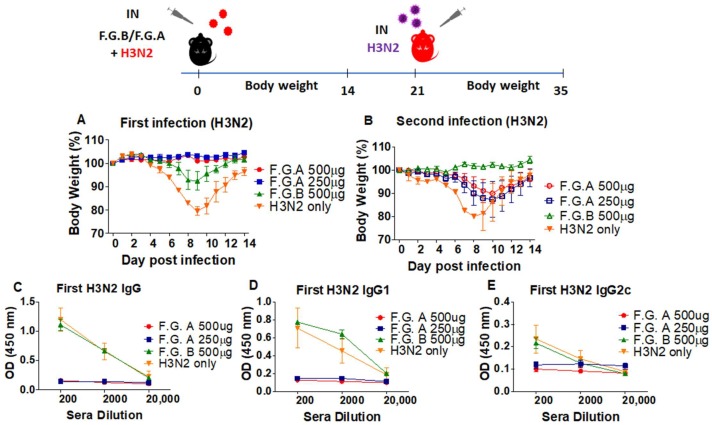
Fermented ginseng sample A showed higher antiviral protective activity against 1.0 LD_50_ H3N2 influenza viruses. Groups of mice (*n* = 5, CD8 T cell-deficient, CD8KO) were intranasally (IN) infected with a mixture of 1 LD_50_ of A/Philippines/82 (H3N2) virus and fermented ginseng sample A or B at different doses (250 and 500 μg). After 14 days of body weight monitoring, the serum sample was collected to detect the levels of IgG and IgG isotypes after the first infection using an ELISA method. The mice that survived from H3N2 virus infection were challenged with homogenous virus (1.5 LD_50_, H3N2). (**A**) Body weight change after the first infection (H3N2) and (**B**) body weight change after the second infection (H3N2). (**C**) H3N2 specific IgG, (**D**) IgG1, and (**E**) IgG2c after the first infection. F.G.A: H3N2 virus + fermented ginseng sample A; F.G.B: H3N2 virus + fermented ginseng sample B; H3N2 only: virus infection without ginseng samples.

CD8^+^ T cell plays an important role in viral clearance and recovery upon influenza virus infection [28]. Because there were no rgH5N1 specific antibodies induced in the previous experiments (Figure 1 and Figure 2), we used CD8 T cell-deficient (CD8 KO) mice (*n* = 3) to investigate whether CD8^+^ T cells were required for conferring protection virus by fermented ginsengs. The fermented ginseng sample A exhibited a similar pattern of protection against A/Philippines/82(H3N2) in CD8 KO mice. Both groups of CD8 KO mice with mixture of H3N2 virus and 500 µg and 250 of F.G.A were fully protected against body weight loss (Figure 3A). The mice from these two groups did not induce virus-specific antibodies (Figure 3C,D,E) and were not able to develop sufficient immunity against follow-up infection with the same H3N2 virus, as evidenced by significant body weight loss (~15%). This is despite the groups showing better protection than naïve mice with H3N2 infection, which resulted in over ~20% weight loss (Figure 3B). Compared to the F.G.A groups, virus inoculation with 500 µg of F.G.B showed less protection against H3N2 virus, resulting in 5–10% body weight loss and the induction of high levels of H3N2 virus-specific antibodies (Figure 3C,D,E). As expected from the levels of IgG antibodies, this 500 µg F.G.B group was well protected during secondary infection H3N2 virus (Figure 3 B). These results suggest that CD8 T cell is not required for fermented ginseng’s antiviral in vivo protection against influenza virus. 

### 3.4. Antiviral Protection by Fermented Ginseng Extract Is Observed Regardless of Influenza Strains and in Severe Immune-Deficient Condition

**Figure 4:**
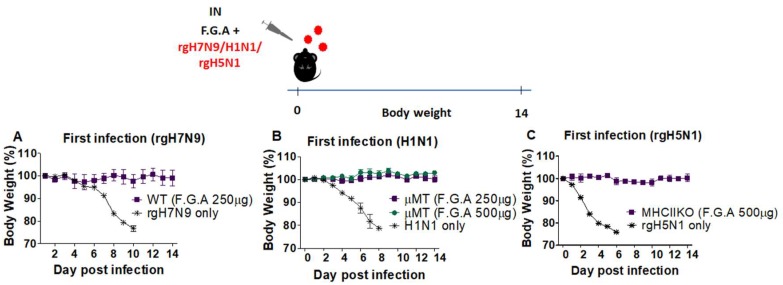
Fermented ginseng sample A had antiviral protective effects against H1N1 or rgH5N1 influenza virus even in severe immune-deficient mice. Groups of wild-type (*n* = 3, C57BL/6) and mutant mice (*n* = 3, B cell-deficient and MHCIIKO) were intranasally (IN) infected with a mixture of virus (2 LD_50_) and fermented ginseng sample A at different doses (250 and 500 μg). Body weight changes were monitored for 14 days after the infection. (**A**) A/Shanghai/2013 (rgH7N9, 2 LD_50_) virus inoculation in wild-type mice; (**B**) A/California/2009 (H1N1) virus inoculation in µMT (B cell deficient) mice; (**C**) A/Vietnam/1203/2004 (rgH5N1) virus inoculation in MHCIIKO mice. F.G.A: fermented ginseng sample A; rgH7N9 only: virus infection without ginseng samples; H1N1 only: virus infection without ginseng samples; rgH5N1 only: virus infection without ginseng samples.

We determined in vivo antiviral protection of fermented ginseng extract against rgH7N9 reassortant virus. As shown in Figure 4A, 250 µg of fermented ginseng sample A protected wild-type C57BL/6 mice that were inoculated with ginseng and rgH7N9 virus. Thus, the antiviral protection by the fermented ginseng samples was observed with rgH5N1 virus, H3N2 virus, and rgH7N9 virus, suggesting universal protection regardless of the type of influenza strain.

Induction of antibodies is known to be the most relevant immune correlate conferring protection against influenza virus. To test whether fermented ginseng sample A inoculation with H1N1 virus would have antiviral protective effects in a B cell-deficient mice, a µMT mouse model was used with a similar protocol as described in previous sections (Figure 4B). The infection of µMT mice (*n* = 3) with the mixture of fermented ginseng sample A and A/California/2009 (H1N1) resulted in complete protection as observed in wild-type mice. Similar results were observed in MHC-II knockout mice. As shown in the Figure 4C, inoculation with rgH5N1 virus and fermented ginseng sample A resulted in complete protection against a lethal virus dose in MHC-II knockout mice. In vivo antiviral protection in B cell-deficient µMT or MHC-II-deficient mice was consistently observed, supporting the conclusion that fermented ginseng samples can inhibit viral replication to a level below the detection limit.

### 3.5. Inoculation of Mice with Fermented Ginseng Extract Samples and Virus Together Inhibits Viral Replication and Lung Inflammation 

**Figure 5:**
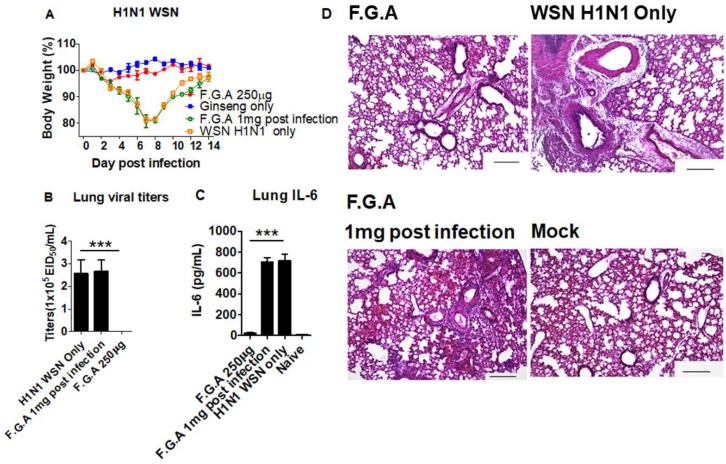
In vivo protection against A/WSN/1933 (H1N1) virus in wild-type BALB/c mice after inoculation with a mixture of fermented ginseng extracts and the virus. Groups of mice (*n* = 5) were infected with a mixture of A/WSN H1N1 virus and fermented ginseng sample A (250 μg). Lung viral titers were determined in the lung extracts at day 6 postinfection. (**A**) F.G.A 250 μg: mixed 250 μg of F.G.A with A/WSN (1× LD_50_) influenza virus; Ginseng only: no WSN virus, just mock infection with 1 mg F.G.A; F.G.A 1 mg postinfection: mice infected with WSN H1N1 first, treated with 1 mg F.G.A one hour postinfection, then treated with 1 mg every 1 h, for five times; H1N1 WSN only: virus infection only. (**B**) Lung viral titers from the mice at day 6 postinfection. (**C**) ELISA for IL-6 in lung. (**D**) Lung histopathology. Mock: no virus, no ginseng; F.G.A: F.G.A 1 mg postinfection; WSN H1N1 only: same as the description above. Magnification: 100×, Scale bars: 50 μm. Ginseng only: inoculation with ginseng, no viruses. *** indicates statistical significance, *p* < 0.0005 between F.G.A and comparing groups.

We further determined the efficacy of antiviral protection in wild-type mice (Figure 5) and CD4KO mice (Supplementary Figures S1 and S2). As expected, the F.G.A 250 µg group showed more effective protection as evidenced by no sign of weight loss (Figure 5A, Supplementary Figure S2A) compared to F.G.B 250 µg group displaying 5–10% weight loss at day 6 postinoculation (Supplementary Figure S2A). Ginseng-only treatment did not have any effects on weight changes in mice without infection. By contrast, the group receiving 1 mg F.G.A treatment postinfection displayed 10–15% weight loss (Figure 5A. F.G.A 1 mg, postinfection), suggesting no protective effects on postinfection. Then, we determined lung viral titers as an in vivo antiviral activity of controlling viral replication at 6 days after inoculation with fermented ginseng extracts and virus (Figure 5B–D). The untreated naïve mice inoculated with WSN H1N1 virus (Figure 5B) and rgH5N1 virus (Supplementary Figure S2B) resulted in the highest viral titers of viral replication in the lung (over 7.4 × 10^5^ titers of egg infectious units). Treatment of F.G.A (1 mg) postinfection resulted in high lung viral titers, similar to the untreated naïve mice (Figure 5B). Mixed virus inoculation with the fermented ginseng sample B lowered lung viral titers to 1.5 × 105 egg infectious units (Supplementary Figure S2B). Nonetheless, viral replication was substantially high in the group of mice with fermented ginseng sample B. Lung viral titers were below the detection limit in the group of mice with F.G.A (Figure 5B, Supplementary Figure S2B), indicating that the fermented ginseng sample A completely inhibited lung viral replication after coinoculation with influenza virus.

To better understand the protective effects on preventing inflammatory disease, we determined proinflammatory cytokine levels in the lung extracts at day 6 postinoculation with ginseng samples and WSN H1N1 or rgH5N1 virus. Wild-type mice infected with WSN H1N1 virus only and ones that received 1 mg of ginseng postinfection showed high levels of inflammatory cytokines (IL-6) in the lung (Figure 5C). Mice infected with a mixture of rgH5N1 virus and fermented ginseng sample B displayed lower but substantial levels of inflammatory IL-6 and TNF-α cytokine in lung and BALF (Supplementary Figure S2C–F). The fermented ginseng sample A group almost completely prevented the induction of proinflammatory cytokines, similar to those in uninfected mice. Therefore, these results suggest that fermented ginseng sample A prevented the induction of inflammatory cytokines by inhibiting influenza virus replication.

Influenza virus infection causes severe lung inflammation with high infiltrating cells in the airways and parenchymal tissues, as shown in our histopathology pictures (WSN H1N1 virus, Figure 5D; rgH5N1 virus, Supplementary Figure S2G). At day 6 postinoculation, lung tissue sections in each group were examined for their histopathology. Wild-type mice inoculated with A/WSN H1N1 virus and then treated with 1 mg fermented ginseng sample A or CD4KO mice inoculated with the mixture of fermented ginseng sample B and virus showed a substantial level of lung inflammation, similar to the mice with naive virus infection (Figure 5D; Ginseng B, Supplementary Figure S2G). The group of mice with fermented ginseng sample A and virus infection prevented the induction of lung histopathology at day 6 postinoculation, similar to the uninfected mouse lungs (F.G.A, Figure 5D; Ginseng A, Supplementary Figure S2G).

### 3.6. The Mice That Survive Primary Virus Inoculation with Fermented Ginseng Extract Samples Develop Immunity against Heterosubtypic Secondary Virus

We found that neither B cells nor CD8^+^ T cells were required for in vivo antiviral activity by fermented ginseng extract samples (Figure 4). Next, we tested whether CD4 T cells would be needed for antiviral protection by fermented ginseng extract samples. A similar pattern of in vivo antiviral protection was observed in CD4 T cell-deficient (CD4KO) mice that were intranasally inoculated with rgH5N1 virus and fermented ginseng extract samples (Supplementary Figure S1A). As expected, the F.G.A (250 µg, 500 µg) CD4KO mouse groups showed protection without weight loss, whereas the F.G.B (250 µg, 500 µg) CD4KO mice displayed moderate weight loss (8–15%), which was lower than the weight loss in untreated CD4KO mice (18–22%).

The mice that survived from the primary virus (rgH5N1) inoculation with fermented ginseng samples were exposed to the secondary H3N2 virus infection with an antigenically different subtype in the absence of additional ginseng treatment (Supplementary Figure S1B). The F.G.B 250 µg treated CD4KO mice that showed an average of 8–9% weight loss during primary infection exhibited heterosubtypic protection against H3N2 virus without weight loss (Figure 5B). This secondary protection against H3N2 virus appears to be due to immunity developed during primary rgH5N1 virus infection. Significant levels of IgG, IgG1, and IgG2c antibodies reactive to rgH5N1 virus were observed in the F.G.B (250 µg, 500 µg) CD4KO mouse groups but not in the F.G.A (250 µg, 500 µg) CD4KO mice (Supplementary Figure S1C–E). It is likely that the doses—250 µg, 500 µg—of F.G.A had more potent antiviral activity, completely inhibiting viral replication to limit the development of immunity. During the secondary infection with H3N2 virus, this 250 μg F.G.A group showed significant body weight loss (15%) and then recovered; this was worse than the F.G.B 500 μg dose group, which saw a 5–6% weight loss and slightly better than the one observed in naïve infection, which showed 18–19% weight loss. By contrast, the 500 μg F.G.A group that was protected against rgH5N1 virus without weight loss displayed severe weight loss of over 20%.

### 3.7. Fermented Ginseng Extract Shows In Vitro Antiviral Activity against Influenza Virus

**Figure 6:**
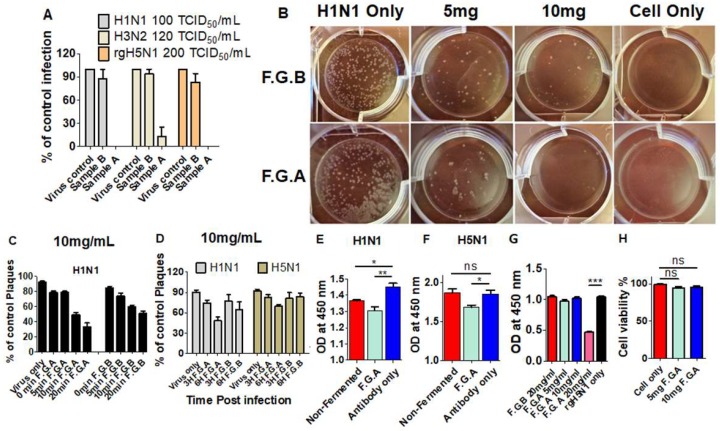
Fermented ginseng extract sample A had antiviral activity against influenza virus in vitro. (**A**) Microneutralization assay. Different strains of viruses (100 TCID_50_ H1N1, 120 TCID_50_ H3N2, and 200 TCID_50_ H5N1) were incubated with 10 mg/mL of F.G.A or F.G.B for 1 h at 37 °C and added to MDCK cells (1 × 10^5^) for 18 h cultures. ELISA was done using primary antibody (Anti-NP) and secondary antibody (IgG-HRP) to determine percentage of growth based on control virus infection without ginseng samples. TCID_50_: Tissue culture infectious dose. (**B**) Plaque assay in MDCK cells. The H1N1 and F.G.A or F.G.B mixture incubated with MDCK cells monolayer for 30 min, then covered with 1.5% agarose to culture for 3–5 days to determine percentage of control plaques. (**C**) Effects of fermented ginseng samples treatment prior to infection on viral growth in plaques. The mixture of H1N1 California/A/2009 and 10 mg/mL of F.G.A or F.G.B was incubated for 0, 5, 10, and 20 min at 37 °C. (**D**) Effects of fermented ginseng samples treatment postinfection on viral growth in plaques. MDCK monolayers were first infected with H1N1 or H5N1 virus and incubated for 1 h at 37 °C. After washing away the virus, 10 mg/mL of F.G.A or F.G.B were added to virus-infected MDCK monolayers for 3 h or 6 h. (**E**,**F**) ELISA for F.G.A-treated viral plates. The 96-wells were coated with H1N1 or H5N1 inactivated virus overnight, then incubated with 10 mg of F.G.A or nonfermented ginseng for 1 h at 37 °C. The primary Anti-H1 HA and Anti-H5 HA and secondary goat anti-mouse IgG were used for ELISA test. (**G**) 8000 TCID_50_ of rgH5N1 virus was incubated with different concentration of F.G.A or F.G.B for 30 min, and neuraminidase activity was determined. (**H**) Cell viability test. 2 mL of EMEM medium (with 5 mg and 10 mg/mL of F.G.A) was incubated with MDCK monolayer for 6 h. Medium–ginseng mixture was removed, and cell viability was counted by trypan blue method. *, **, *** symbols indicate statistical significance, *p* < 0.05, *p*< 0.01 and *p* < 0.001 between the comparing groups. ‘ns’ indicates no significance.

Since fermented ginseng extract has in vivo antiviral protection against the influenza virus, we determined whether this ginseng sample has antiviral activity in vitro. The fermented ginseng sample B (10 mg/mL) only inhibited 10–15% of viral infection of H1N1, H3N2, and rgH5N1, as determined by the microneutralization method (Figure 6A). In comparison, 10 mg/mL of sample A neutralized most of the virus of H1N1, H3N2, and rgH5N1, so influenza virus could not properly replicate in MDCK cells.

The anti-influenza virus activities of fermented ginseng extract against H1N1 and rgH5N1 were further examined by a plaque assay. As shown in Figure 6B, 10 mg/mL fermented sample A reduced more than 95% of plaque-forming units in H1N1 virus, while fermented sample B reduced about 70% of the plaques.

Different incubation time of H1N1 virus with sample A and B prior to infection was tested (Figure 6C). In the figure, 0 min and 5 min of incubating time of sample A reduced 20–25% of plaques. Extended incubation time of 20 min reduced more than 60% of forming plaques. Although the sample B had less inhibitive ability, it also reduced plaques as the incubation time was extended.

We also investigated whether fermented ginseng extract would exhibit inhibitory effects on influenza virus plaque formation upon treatment after MDCK cells had been infected with influenza virus. Approximately 25% and 50% reduction in plaques were observed with 3 and 6 h incubation, respectively, of 10 mg/mL sample A with MDCK cells that were infected with H1N1 or rgH5N1 virus prior to ginseng sample treatment (Figure 6D). Fermented ginseng sample B treatment postinfection was less effective in reducing viral plaques (25–35%) than sample A.

Next, we determined the effects of fermented ginseng extract on hemagglutination and neuraminidase activity and on antibody binding to the virus. We found 10 mg/mL of F.G.A significantly decreased the capture ability of primary antibody of anti-H1 HA or anti-H5 HA to the coated H1N1 or H5N1 on 96-well plate, respectively (Figure 6E,F). Meanwhile, high doses (10 mg/mL) of fermented ginseng sample A (but not sample B) prevented 4 HA units of H1N1 and H5N1 HA-mediated hemagglutination activity (Supplementary Figure S3). As shown in Figure 6G, 20 mg/mL of fermented ginseng sample A exhibited inhibitory effects on neuraminidase activity of rgH5N1 virus, whereas ginseng sample B did not show such neuraminidase inhibitory activity. 

We also tested the cytotoxicity of F.G.A in vitro and in vivo. Different concentrations of F.G.A were added to 2 mL of EMEM medium at final concentrations of F.G.A 5 mg and 10 mg, respectively. As shown in Figure 6H, 5 mg and 10 mg/mL of F.G.A had no impact on cell viability. Meanwhile, mice that were intranasally treated with 1 mg of F.G.A maintained the bodyweight during 14 days of monitoring (Figure 5A, ginseng only).

## 4. Discussion

This study presents data demonstrating that fermented ginseng extract with more saponin component ginsenosides (F1, F2, PPT, Rh2, PPD) increased survival rates and protected the mice against body weight loss when coinoculated with influenza virus and ginseng samples. Fermented ginseng sample A (F.G.A) and sample B (F.G.B) presented different anti-influenza effects, possibly due to the different saponin components. Compared to F.G.B, F.G.A showed more potent antiviral activity against rgH5N1, H3N2, H1N1, and rgH7N9 influenza virus, not only in wild-type C57BL/6 and BALB/c mice but also in CD4 T cell-deficient mice, CD8 T cell-deficient mice, B cell-deficient mice and MHC-II-deficient mice with defects in CD4 and MHC II-antigen presentation. In F.G.A-treated group (250 µg or higher dose), there were no virus replication and inflammation in the lung, while the mice with F.G.B still had substantial levels of viral loads and inflammation due to the influenza virus infection. It is assumed that the dose (250, 500 μg) of F.G.A conferred in vivo protection, inhibiting viral replication to a low level and preventing disease (no weight loss). It also limited the IgG production but allowed low viral replication, priming cellular immunity such as cross-protective T cell responses. During the secondary infection with the homologous virus (Figure 1) or H3N2 virus (Figure 3), the 250 μg and 500 μg F.G.A groups showed approximately 15–20% body weight loss and then recovered. This is a similar weight loss pattern as in the mock control infection, although the 250 μg and 500 μg F.G.A groups recovered better. It is assumed that F.G.A-treated groups mediated a low degree of secondary protection by a low level of (non-neutralizing) cellular immunity that had been induced during the primary infection despite the absence of detectable levels of virus-specific IgG antibodies. The F.G.A low dose concentration below 20 µg allowed the replication of viruses to sufficient high levels, causing significant weight loss (disease), and stimulated the induction of high levels of virus-specific antibodies. Mice infected with a mixture of rgH5N1 virus and F.G.B displayed lower levels of inflammatory cytokines (IL-6, TNF-α) in lung and in BALF compared to virus infection of the control mice. Meanwhile, F.G.A almost completely prevented the induction of proinflammatory cytokines, similar to those in uninfected mice. A similar pattern of protection against intranasal infection with mixtures of F.G.A and the virus was observed in both wild-type and CD4 knockout mice (Figure 5 and Supplementary Figure S1). Consistent with the approaches and results in our studies, extracts of other plants, such as Echinacea purpurea, have also been shown to have anti-influenza effects at a similarly low dose (50 µg); direct contact between Echinacea purpurea and virus was found to be required for maximum antiviral effect [29]. In another relevant study [30], influenza virus (1 × 10^3^ EID_50_) was preincubated with equal volumes of ginseng extract, Rb1, or other ginsenosides (2 mg/mL) at 37 °C for 1 h. The virus and compound mixture were administered to naïve animals intranasally under anesthesia, reporting the antiviral activity of Rb1 ginsenoside. Rb1 was shown to interact with viral hemagglutinin protein, preventing viral attachment to the 2–3 sialic acid receptors on the target cells [30]. 

Fermented ginseng samples appeared to exhibit more effective antiviral protection effects on influenza virus in vivo than in vitro. The data in this study suggest that direct contact between F.G.A and the virus resulted in antiviral effects in vivo, regardless of host adaptive immunity, such as CD4 and CD8 T cells. We did not observe protective effects of fermented ginseng extract when it was used to treat mice that had already received infection. Treatment of fermented ginseng extract before influenza infection via oral route [30] did not result in any substantial protection against influenza either. In in vitro experiments, F.G.A showed effects on inhibiting virus plaque formation and suppressing HA and NA activities, which likely partially contributed to the in vivo protection observed. A 100 µg dose of F.G.A could effectively inhibit influenza virus replication in mice. It is possible that incubation of ginseng samples of ginsenosides compounds with influenza virus might induce conformational changes in viral surface proteins and reduce their infectivity to mammalian cells [30]. After infection of influenza virus, macrophages and neutrophils were rapidly recruited in the lung [31]. A high dose range (250–500 µg) of fermented sample A might have neutralized most of the influenza viruses inoculated in a mixed form, and innate immune cells might have cleaned the remaining viruses. This protection through a mixture of F.G.A and virus in naïve mice was independent of adaptive immune components, such as CD4 T cells, CD8 T cells, MHCII, and B cells. There was no antibody response detected in the mice that received a high dose of fermented ginseng sample A mixed with influenza virus. The high dose F.G.A-protected mice without weight loss did not develop sufficient immunity and were not protected after secondary influenza virus infection. It is speculated that the dose (250 μg) of F.G.A in CD4 KO mice conferred in vivo protection, inhibiting primary viral replication (rgH5N1 virus) to a low level and preventing disease. It also limited the IgG production but allowed low viral replication, priming cellular immunity such as cross-protective CD8 T cell responses. During the secondary infection with H3N2 virus, this 250 μg F.G.A group (no detectable IgG antibodies against rgH5N1) showed approximately 15% body weight loss and then recovered, which was worse than the F.G.B 500 μg dose (IgG antibodies against rgH5N1) that caused 5–6% weight loss. The 250 μg F.G.A group was only slightly better than the one observed in naïve infection. The 250 µg F.G.B treatment group or mock treatment with infection, which experienced significant weight loss during the first infection and developed rgH5 virus-specific IgG antibodies, were fully protected against H3 virus in mice. There are multiple parameters in this observation of cross-protection in primary survival mice during the secondary infection. There might be cross-protective neutralizing IgG antibodies in mice that survived the primary infection. Cross-protection during the secondary infection is a well-known phenomenon in mice that survive infection with pathogenic influenza viruses. In previous studies, the mice that survived pathogenic influenza virus infection and experienced substantial weight loss during primary infection were shown to have heterosubtypic immunity through the induction of cross-reactive T cell and B cell responses [32,33,34,35,36]. The heterosubtypic protection was observed in the mice that survived primary infection even in a condition with T cell depletion during the secondary infection with an antigenically different virus [37]. Ginseng was shown to have anti-inflammatory activity, conferring the host with resistance to inflammatory disease against viral infections [38,39], which suggests a possibility of modulating inflammatory innate immune responses by F.G.B.

Moreover, antiviral activity of fermented ginseng was observed in a dose-dependent manner in vivo. High concentration of F.G.A (above 100 µg) related with full protection—no antibody response (Figure 2, F.G.A 500 µg and 100 µg); low concentration of F.G.A (below 20 µg) related with no protection—antibody response (Figure 2, F.G.A 20 µg). It might have allowed low levels of virus replication in a threshold dose F.G.A (50 µg) and produced the antibody response (Figure 2, F.G.A 50 µg). It has also been reported that ginsenosides interact with viral hemagglutinin proteins and prevent viral attachment with sialic acid receptors [30]. It might be possible that the active ginsenosides in fermented ginseng bind to hemagglutinin and thus prevent the infections. The data from the interference of binding primary anti-HA antibody suggest that F.G.A components might directly interact with virus HA proteins. In addition, F.G.A also inhibited HA activity when incubated in vitro with influenza virus (Supplementary Figure S3). Normally, neuraminidase plays an important role in budding and spreading of influenza virus. Neuraminidase also facilitates the entry of influenza virus into the human airway epithelium cells [40]. These in vitro test results indicate that inhibitory effects on HA and NA activity might have partially contributed to in vivo antiviral protection by fermented ginseng samples.

It is of interest to investigate which ginseng saponin ginsenosides function as the real effective antiviral component. After fermentation, the fermented ginseng sample A and B were both found to contain higher levels of compound K and active ginseng saponin components ginsenosides (PPT, Rh2, PPD, F1, F2). It is speculated that combined effects of multiple substances in fermented ginseng samples might have such antiviral effects against influenza viruses with diverse subtypes. High concentrations (>10 mg/mL) of the fermented ginseng extract samples in in vitro antiviral test assays would not be in a range of an efficacious therapeutic level. Administration of mixed ginseng samples and viruses would not have much clinical relevance. In other aspects, this study focused on in vivo protective effects on influenza virus by fermented ginseng samples after direct contact with the virus. Inoculation with mixed pathogenic virus and fermented ginseng samples at appropriate dose resulted in protection in naïve mice without displaying weight loss during primary infection. In addition, the mice that survived or were protected during primary infection could develop immunity against homologous and different strains during the secondary infection. This finding could be applied to develop a safer live vaccine formulation for high-risk populations. Further studies on the safety, immunogenicity, and efficacy aspects should be performed.

Translating into clinical relevance, intranasal (spray) treatment with fermented ginseng samples at the site of virus entry during severe epidemic or pandemic seasons might have some protective benefits. Further studies remain to be done to uncover the anti-influenza virus mechanisms of fermented ginseng samples.

## 5. Conclusions

In the present study, we found that fermented ginseng extracts containing ginsenosides (PPT, PPD, Rh2, and Compound K) displayed more powerful antiviral effects against influenza viruses than nonfermented ginseng extracts. The antiviral protective effects were observed regardless of influenza virus strains, including various subtypes of H1N1, H3N2, H5N1, and H7N9. Mice that were inoculated with a moderate dose of fermented ginseng extract samples and a lethal dose of virus were protected against weight loss with 100% survival rates during primary infection as well as developed immunity against secondary viral infection.

## Figures and Tables

**Table 1:** Components of ginseng samples (mg/g).

Ginsenosides	Red Ginseng Extracts	Fermented Ginseng A	Fermented Ginseng B
Rg1	1.36	0.60	1.27
Re	1.43	0.49	0.87
Rf	1.02	0.29	0.13
Rb1	6.86	0.95	2.74
Rc	2.76	0.04	0.05
Rg2	1.03	0.65	0.25
Rh1	1.02	0.58	0.19
Rb2	2.46	0.02	0.26
Rb3	0.57	0.01	0.02
F1	-	0.28	0.17
Rd	0.82	0.62	0.75
F2	-	0.55	0.47
Rg3	1.51	0.62	0.54
PPT	-	0.88	0.33
CK	-	4.19	1.69
Rh2	-	1.49	0.43
PPD	-	2.13	0.93
Total ginsenosides	21.0 mg/g	14.39 mg/g	11.09 mg/g

CK: compound K, PPD: protopanaxadiol, PPT: protopanaxatriol

## Supplementary Materials

The following are available online at http://www.mdpi.com/1999-4915/10/9/471/s1, Figure S1: *In vivo* primary and secondary protection against rgH5N1 and H3N2 virus in CD4 deficient mice, Figure S2: CD4 KO mice after infection with rgH5N1 influenza virus mixed with fermented ginseng sample A Lung viral replication are protected by clearing lung viral titers, Figure S3: Inhibition of hemagglutination activity in vitro by fermented ginseng extracts.
