# Supplementary material for: Antiviral Activity of Fermented Ginseng Extracts against a Broad Range of Influenza Viruses
Source: Viruses. 2018 Sep 1;10(9):471. doi: 10.3390/v10090471 (PMC6164659; doi:10.3390/v10090471)
Supplement: Supplementary file 1 [file viruses-10-00471-s001.pdf]

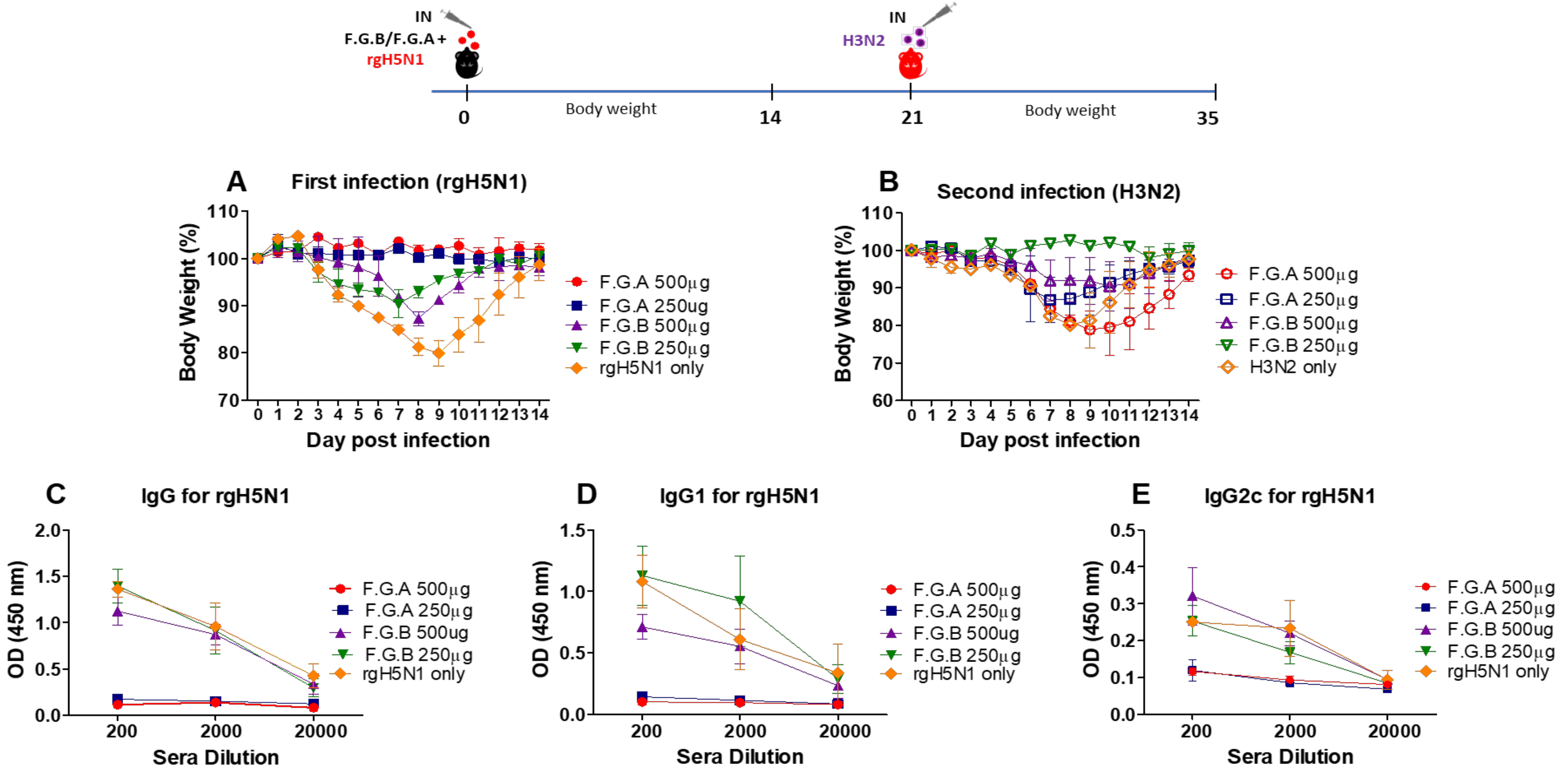

Supplementary Fig.1. *In vivo* primary and secondary protection against rgH5N1 and H3N2 virus in CD4 deficient mice. Groups of CD4 knockout mice (CD4KO, n=5) were infected with a mixture of 1.2LD_{50} A/Vietnam/1203/2004 (rgH5N1) and fermented ginseng sample A or B at different doses (250μg and 500μg). After 14 days of body weight monitoring, the serum sample was collected to detect the levels of IgG and IgG isotypes after first infection. The survived mice from rgH5N1 virus infection was challenged again with 1.0LD_{50} of A/Philippines/82 (H3N2) virus. (A) Body weight change after first infection (rgH5N1). (B) Body weight change after second infection (H3N2). rgH5N1 specific IgG (C), IgG1 (D), and IgG2c (E). F.G.A: rgH5N1 virus + fermented ginseng sample A, F.G.B: rgH5N1 virus + fermented ginseng sample B, rgH5N1 only: virus infection without ginseng samples, H3N2 only: virus infection without ginseng samples.

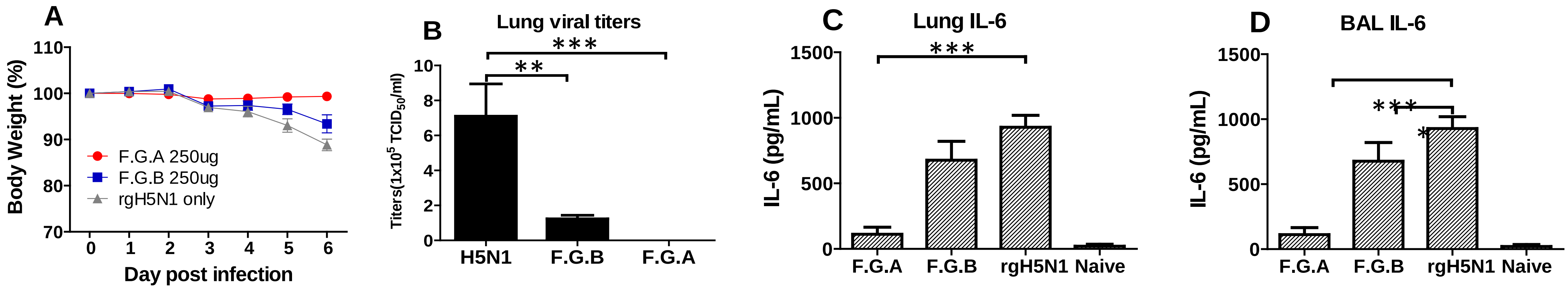

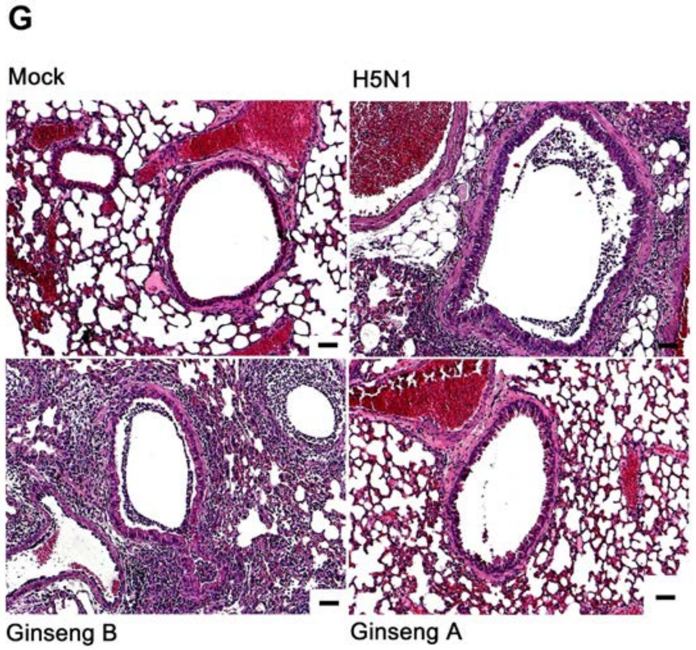

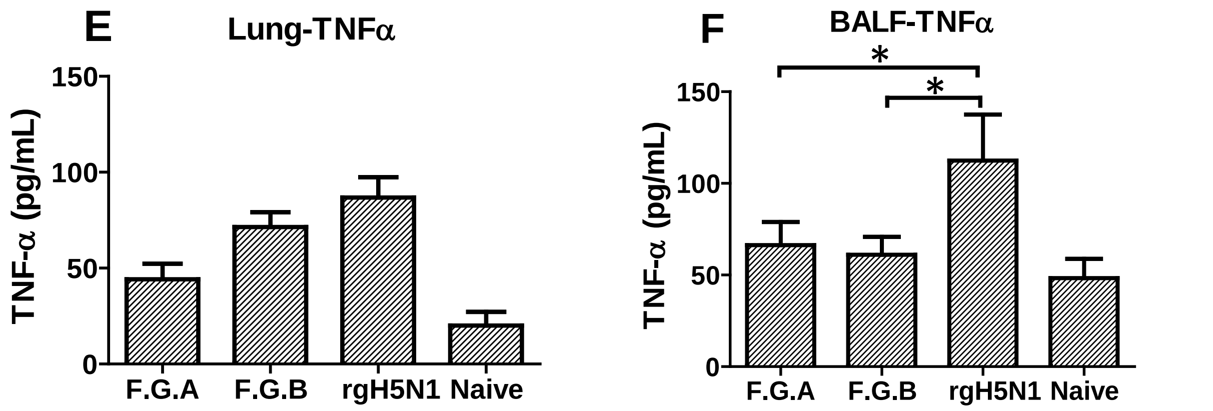

Supplementary Fig.2 CD4 KO mice after infection with rgH5N1 influenza virus mixed with fermented ginseng sample A Lung viral replication are protected by clearing lung viral titers. (A-B) Groups of mice (n=5, CD4 T cell-deficient, CD4KO) mice were infected with a mixture of 1.5 LD_{50} rgH5N1 virus and fermented ginseng sample A or B (250ug). Lung viral titers were determined in the lung extracts at day 6 post infection. F.G.A: Fermented ginseng sample A. F.G.B: Fermented ginseng sample B, H5N1 only: virus infection without ginseng samples. (C-F) Cytokines were determined in the lung and bronchoalveolar lavage fluid (BALF). (G) Lung histopathology. F.G.A: rgH5N1 virus + fermented ginseng sample A, F.G.B: H5N1 virus + fermented ginseng sample B, H5N1: virus infection without ginseng samples. Mock: No virus, No ginseng. Magnification:100×, Scale bars: 50 μm.

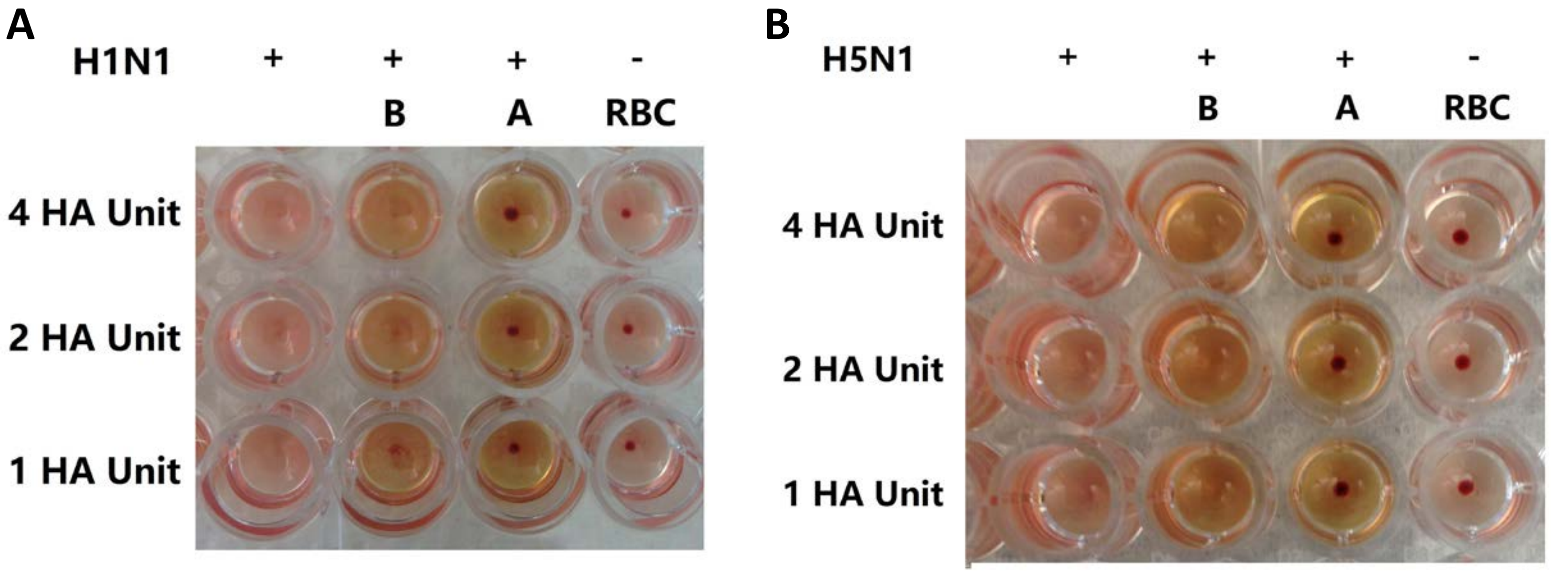

Supplementary Figure. 3 Inhibition of hemagglutination activity in vitro by fermented ginseng extracts A. B. Hemagglutinin inhibition test. Incubated 25ul of 1, 2, 4 HA unit of H1N1 virus with another 25ul of 10mg/ml of F.G.A or F.G.B for 1h at 37°C, then mixture was treated by 0.5% chicken blood cells.
